# Inactivation of *Pseudomonas aeruginosa* biofilms by thymoquinone in combination with nisin

**DOI:** 10.3389/fmicb.2022.1029412

**Published:** 2023-01-19

**Authors:** Hong Chen, Peng-Cheng Ji, Yue-Heng Qi, Shi-Jin Chen, Chang-Yao Wang, Yu-Jie Yang, Xin-Yu Zhao, Jin-Wei Zhou

**Affiliations:** ^1^Luoyang Key Laboratory of Organic Functional Molecules, College of Food and Drug, Luoyang Normal University, Luoyang, China; ^2^School of Food and Biological Engineering, Xuzhou University of Technology, Xuzhou, China

**Keywords:** *Pseudomonas aeruginosa*, virulence, biofilm, transcriptomics, metabonomics

## Abstract

*Pseudomonas aeruginosa* is one of the most important foodborne pathogens that can persist in leafy green vegetables and subsequently produce biofilms. In this study, the synergistic effect of thymoquinone and nisin in reducing biofilm formation of *P. aeruginosa* on lettuce was evaluated, and their anti-virulence and anti-biofilm mechanisms were also investigated. At concentrations ranging from 0.5 to 2 mg/ml, thymoquinone inhibited the production of autoinducers and virulence factors, and enhanced the susceptibility of *P. aeruginosa* biofilms to nisin as evidenced by the scanning electron microscopy and confocal laser scanning microscopy. Integrated transcriptomics, metabolomics, and docking analyses indicated that thymoquinone treatment disrupted the quorum sensing (QS) system, altered cell membrane component, and down-regulated the expressions of genes related to virulence, efflux pump, and antioxidation. The changed membrane component and repressed efflux pump system enhanced membrane permeability and facilitated the entrance of nisin into cells, thus improving the susceptibility of biofilms to nisin. The dysfunctional QS and repressed antioxidant enzymes lead to the enhancement of oxidative stress. The enhanced oxidative stress disrupted energy metabolism and protein metabolism and ultimately attenuated the virulence and pathogenicity of *P. aeruginosa* PAO1. Our study indicated that thymoquinone has the potential to function as a QS-based agent to defend against foodborne pathogens in combination with nisin.

## 1. Introduction

*Pseudomonas aeruginosa* is a notorious opportunistic pathogen associated with the infection of humans through extensively contaminated foods such as meat, seafood, dairy products, and vegetables (Gomes Neto et al., 2012). Among these illness-causing vegetables, lettuce is an important concern (Allydice-Francis and Brown, 2012). Consuming raw lettuce that have been contaminated by *P. aeruginosa* could have serious impact on human health (Allydice-Francis and Brown, 2012). Synthetic and natural preservatives are the most commonly used means to control lettuce contamination induced by *P. aeruginosa*. However, the extensive use or abuse of preservatives has led to the enhanced drug resistance of this foodborne bacterium. Consequently, it is urgent to develop novel alternative measures to control food contamination without causing survival pressure as preservatives.

*P. aeruginosa* has the potential to attach to the surface of lettuce leaves and to develop biofilms during the cultivation, storage and processing steps. Biofilms have been regarded as a vital ability employed by *P. aeruginosa* to escape pressures derived from preservatives during food contamination (Yin et al., 2022). Biofilms are bacterial clusters, in which bacteria are encapsulated by extracellular matrix consisting of eDNA, exopolysaccharides and proteins that prevent preservatives from getting inside cells (Zhou et al., 2021). *P. aeruginosa* formed biofilms on lettuce leaves during lettuce processing, resulting in serious economic losses related to food contamination (Balabanova et al., 2020). Biofilm formation of *P. aeruginosa* in lettuce products enhances the danger of lettuce contamination and has attracted extensive attention in food industry. Therefore, continuous efforts have been made to block *P. aeruginosa* biofilm formation in lettuce processing (Yin et al., 2022).

A lot of synthetic and natural products have the potential to inhibit biofilm formation of *P. aeruginosa* (O'Loughlin et al., 2013; Gutierrez-Barranquero et al., 2015). However, the constrained use of these products in food industry and mammalian cells has promoted the exploitation of new natural biofilm inhibitors with broad applications (Gopu et al., 2015). Nisin, a widely used preservative in food industry, has powerful anti-biofilm activity against Gram-positive bacteria (Hossain et al., 2022). However, it shows weak anti-biofilm activity against Gram-negative bacteria including *P. aeruginosa* (Field et al., 2016a). Therefore, exploring natural products that can enhance the anti-biofilm potential of nisin is necessary. Thymoquinone, a predominant quinone compound from black seed oil, has traditionally been used as antioxidant and antimicrobial agent in food (Khader et al., 2009). However, the synergistically inhibitory effect of thymoquinone with nisin on biofilm formation of *P. aeruginosa* on lettuce has not been reported. In addition, the anti-virulence and synergistic mechanisms of thymoquinone against *P. aeruginosa* have not been deeply revealed. Here, the transcriptome sequencing, docking simulation, and ^1^H NMR-based metabolomics were adopted to investigate the anti-virulence and synergetic mechanisms of thymoquinone against *P. aeruginosa*.

## 2. Materials and methods

### 2.1. Minimum inhibitory concentration and growth curve

*P. aeruginosa* PAO1 (ATCC 15692) was cultivated in Luria-Bertani (LB, Sangon Biotech, Shanghai, China) at 37°C unless otherwise specified. Nisin and thymoquinone were purchased from Shanghai Yuanye Biotech (Shanghai, China). Nisin and thymoquinone were dissolved in dimethyl sulfoxide (DMSO) and their MICs were determined by twofold serially diluted method with an inoculum of 1–5 × 10^5^ CFU/ml (Zhou et al., 2018b). For growth curve, 0.1% overnight cultures of *P. aeruginosa* PAO1 were inoculated into LB broth supplemented with different concentrations of thymoquinone (0.5–4 mg/ml). DMSO was used as the negative control. After cultivation at 37°C for 24 h, growth was measured by reading OD_600_. Each treatment was carried out three times independently.

### 2.2. Analysis of autoinducers production

The impact of thymoquinone on the production of C4-HSL and PQS was determined by inoculating *P. aeruginosa* PAO1 into fresh LB medium to obtain a final concentration of 5 × 10^5^ CFU/ml supplemented with thymoquinone at concentrations ranging from 0.5 to 2 mg/ml. After incubation at 37°C for 24 h, the sterile supernatant was polled, freeze-dried, and re-dissolved with methanol. The presence of C4-HSL and PQS were identified through liquid chromatography–tandem mass spectrometry (LC–MS/MS) according to the retention time of standard chemicals and their MS/MS^2^ spectra, and their levels were quantified according to the peak area relative to the standard chemicals (Zhou et al., 2019b).

### 2.3. Virulence factors inhibition

Overnight cultures of *P. aeruginosa* PAO1 (10^5^ CFU/ml in LB) were incubated with various concentrations of thymoquinone (0.5–2 mg/ml) at 37°C for 24 h. DMSO and the QS inhibitor carvacrol (Tapia-Rodriguez et al., 2017; 1 mg/ml) were used as the negative and positive control, respectively. After incubation, 150 μl of supernatant was mixed with 250 μl of 0.3% azocasein. The mixture was cultivated at 37°C for 4 h followed by the addition of 1.2 ml of 10% trichloroacetic acid. Subsequently, 1.2 ml of 1 M NaOH was supplemented to terminate the reaction. Protease activity was measured by reading OD_440_ (Wu et al., 2016).

For elastase analysis, 100 μl of supernatant was mixed with 900 μl of assay buffer (1 mM CaCl_2_, 100 mM Tris, pH 7.5) containing 20 mg of elastin Congo red as described previously (Ohman et al., 1980). After incubation at 37°C for 3 h, the mixture was centrifuged and the supernatant was collected for optical-density reading at 495 nm.

For pyocyanin quantification, 5 ml of supernatant was extracted with 3 ml of chloroform. The organic layer was re-extracted with 1 ml of 0.2 M HCl. After centrifugation, the color intensity was measured at 520 nm (Chen et al., 2017).

Rhamnolipids were determined as described by Kim et al. (2015). Briefly, 300 μl of supernatant was extracted with 600 μl of diethyl ether. The ether phase was evaporated and residuals were eluted in 100 μl of deionized water followed by the addition of 900 μl of orcinol solution. After 30-min boiling, the absorbance of the cooled mixture was read at 421 nm.

Alginate was quantified according to Packiavathy et al. (2014) with minor modification. Briefly, 70 μl of supernatant was mixed with 600 μl of boric acid/H_2_SO_4_ (4:1, v/v) followed by the addition of 20 μl of 0.2% carbazole solution. The mixture was incubated at 55°C for 30 min. Alginate was quantified by measuring OD value at 530 nm.

Lipase activity was assayed as described by Achouri et al. (2021). Briefly, 10 μl of thymoquinone or DMSO-treated bacteria cells were added to 1.5% agar medium containing 2% Tween 20. Lipase activity was quantified by measuring the diameter of the hale after incubation at 30°C for 96 h.

Chemotaxis assay was assayed by inoculating 2 μl overnight cultures of *P. aeruginosa* PAO1 on one side of the ATGN medium (without glucose) with various doses of thymoquinone as described previously (Ding and Christie, 2003). One strip filter paper soaked with glucose was placed 4 cm aside from the bacterial drop, and then incubated at 37°C for 48 h. The chemotaxis diameter was determined. Swimming and swarming motilities were assayed as described by Sheng et al. (2015) with minor modification. Briefly, 2 μl overnight cultures of *P. aeruginosa* PAO1 was inoculated with different concentrations of thymoquinone at the center of the swimming (1% tryptone, 0.5% NaCl, 0.3% agar) and swarming medium (1% tryptone, 0.5% NaCl, 0.5% glucose, 0.3% agar), respectively. Carvacrol (1 mg/ml) and DMSO were used as the positive and negative control, respectively. The plates were incubated at 37°C for about 48 h and then the migration zones were measured.

### 2.4. Biofilm inhibition assay on microplates

The enhanced effect of thymoquinone on biofilm formation with the addition of nisin was determined by the crystal violet staining method using 96-well polystyrene plates (Cho et al., 2013). Briefly, overnight cultures of *P. aeruginosa* PAO1 were inoculated into TSB medium (Hopebio, Qingdao, China; 10^5^ CFU/ml) containing thymoquinone and nisin (1 mg/ml). After incubation at 37°C for 24 h, the suspensions were removed and the remaining biofilms were washed with deionized water to remove planktonic cells. The sessile cells were stained with 0.05% crystal violet, solubilized in 95% ethanol, and then quantified by measuring OD_570_ (Biotek Elx800, Winooski, VT, United States). Each treatment was carried out three times independently.

For visual observation of the formed biofilms, samples were subjected to scanning electron microscopy (SEM, JSM6360, JEOL, Japan) and confocal laser scanning microscopy (CLSM, Zeiss LSM 700, Carl Zeiss, Jena). Briefly, biofilms were incubated in 24-well microplates with round glass coverslips as described above. After cultivation, coverslips were washed with sterile water and dehydrated with graded ethanol. The freeze-dried samples were coated with gold and then observed with SEM. For CLSM analysis, samples were stained with acridine orange (AO) and ethidium bromide (EB; 1:1) for 15 min and then washed with sterile water to remove excess dye. The stained biofilms were subjected to CLSM using a × 63/1.4 numerical aperture oil objective. Three-dimensional reconstructions were analyzed by the ZEISS confocal software (ZEN, 2012) (Zhou et al., 2019b).

### 2.5. Effect of thymoquinone and nisin on biofilm formation on lettuce leaves

The inoculation of *P. aeruginosa* PAO1 on lettuce leaves was performed using the method as established by Hussain et al. (2019) with minor modifications. Briefly, overnight cultures of *P. aeruginosa* PAO1 were 0.1% (10^5^ CFU/ml) inoculated into LB medium containing thymoquinone (1 and 2 mg/ml) and nisin (1 mg/ml). After incubation at 37°C at 180 rpm for 18 h, bacterial cells were collected by centrifugation. Cells were washed with sterile PBS and then re-suspended to obtain a final concentration of 10^8^ CFU/ml. A total of 20 μl of suspensions were pipetted onto the surface of the lettuce leaf pieces. Each piece of lettuce leaf was placed in separate Petri dishes and incubated at 37°C for 2 days. The formed biofilms were rinsed with sterile PBS to remove unattached cells and then transferred to a sterile tube. Dextranase (5 U, D8144-Sigma-Aldrich, USA) was supplemented to digest the exopolysaccharides followed by sonication for 30 s (37 Hz, KQ-250, Kunshan Ultrasonic Instruments Co., Ltd., China). The number of CFU/biofilm was calculated by plating the resulting dilutions on LB agar at 37°C overnight (Brackman et al., 2011).

### 2.6. RNA extraction and sequencing

DMSO and thymoquinone-treated *P. aeruginosa* PAO1 were selected for RNA extraction. Total RNA was extracted using TRIzol® Reagent according the manufacturer’s instructions (Invitrogen) and genomic DNA was removed using DNase I (TaKara). RNA quality was determined by 2,100 Bioanalyser (Agilent) and quantified using the ND-2000 (NanoDrop Technologies). Only high-quality RNA sample (OD260/280 = 1.8 ~ 2.0, OD260/230 ≥ 2.0, RIN ≥ 6.5, 28S:18S ≥ 1.0, ≥100 ng/μl, ≥2 μg) was used to construct sequencing library. RNA-seq transcriptome library was prepared following TruSeqTM RNA sample preparation Kit from Illumina (San Diego, CA) using 2 μg of total RNA. The paired-end RNA-seq sequencing library was sequenced with the Illumina HiSeq × TEN (Illumina, San Diego, CA; 2 × 150 bp read length).

High quality reads in each sample were mapped to the *P. aeruginosa* PAO1 reference genome using Bowtie2 (version: 2.4.5). The FPKM (Fragments Per Kilpbase Million) and TPM (Transcripts Per Million) eliminated the influence of different gene length and sequencing discrepancy on the calculation of gene expression. Differentially expressed genes (DEGs) between the DMSO and thymoquinone-treated groups were identified with DESeq2. The criteria for selecting samples were: *p* < 0.05 and FC (fold change) > 2. GO enrichment analysis and KEGG pathway analysis were used to clarify the role of differentially expressed genes. Significant enrichment of DEGs in GO terms and metabolic pathways was marked by a *p*-value (Bonferroni correction) of less than 0.05.

### 2.7. ^1^H NMR-based analysis of intracellular metabolites

*P. aeruginosa* PAO1 was cultivated with DMSO or thymoquinone as mentioned above. Each treatment was carried out six times independently. After cultivation, the cultures were centrifuged at 10,000 rpm for 10 min and the cell pellet was harvested. Cells were washed with PBS and then added with 3.8 ml of precooled methanol/water (1/0.9, v/v) and 4 ml of chloroform for extraction. The dried samples were reconstituted with D_2_O phosphate buffer and then shifted into NMR tubes for NMR analysis (Bruker AVANCE, 500 MHz; Chen et al., 2017). The metabolite assignment was performed by referring publicly accessible metabolomics databases as previously described (Chen et al., 2017).

### 2.8. Reactive oxygen species and H_2_O_2_ analysis

ROS was assayed by inoculating 6-carboxy-2′,7′-dichlorodihydrofluorescein diacetate (1 mM) into the pre-treated bacterial cultures. DMSO was used as the negative control and the concentration of thymoquinone was 2 mg/ml. After 30-min incubation at 30°C, cells were re-suspended with PBS and then determined at 485 nm for excitation and 525 nm for emission (Zhou et al., 2019b). For H_2_O_2_ analysis, cells were harvested and re-suspended with PBS. DMSO was used as the negative control and the concentration of thymoquinone was 2 mg/ml. H_2_O_2_ was released from the cell and equilibrated in the solution. After 1-min centrifugation at 6,000 *g*, the suspension was selected for H_2_O_2_ analysis by the horseradish peroxidase-scopoletin approach (Gonzalez-Flecha and Demple, 1997). Each treatment was carried out three times independently.

### 2.9. Permeability of plasma membrane

Permeability of plasma membrane was evaluated using the method established by Tao et al. (2011). Overnight cultures of *P. aeruginosa* PAO1 were centrifuged at 10,000 *g* for 5 min. The supernatant was removed and cells were gathered. The gathered cells were washed with sterile PBS and then re-suspended with it to obtain a final concentration of 1–5 × 10^5^ CFU/ml. Thymoquinone was added to the suspensions to get an ultimate dose of 2 mg/ml. The same amount of DMSO was used as the negative control. The mixtures were incubated at 37°C for 24 h and the electric conductivity was measured using a conductivity meter (DDS-307, Shanghai Yueping Scientific Instrument Co., Ltd., Shanghai, China).

### 2.10. Real-time quantitative PCR analysis

*P. aeruginosa* PAO1 was grown in LB medium supplemented with or without 2 mg/ml of thymoquinone at 37°C at 180 rpm for 24 h. DMSO was used as the negative control. After incubation, cells were collected by 10-min centrifugation at 10,000 *g* at 4°C and then washed with precooled sterile PBS. Total RNA was extracted using TRIzol® Reagent according the manufacturer’s instructions (Invitrogen) and genomic DNA was removed using DNase I (TaKara), and 10 DEGs involved in quorum sensing, virulence factors, TCA cycle, and drug resistance were selected to validate the RNA-Seq results using RT-qPCR analysis. The reference gene *rpsL* was selected for normalization and the primers were listed in Table 1. The RT-qPCR was carried out in a 20 μl system using MonAmpTM SYBR Green qPCR mix (Monad, China) as recommended by the manufacturer. The reactions were performed using LightCycler 96 Instrument (Roche Diagnostics, United States) with the following cycle parameters: 95°C for 30 s, followed by 40 cycles of 95°C for 5 s, 60°C for 30 s, and 95°C for 15 s. All treatments were performed in triplicate and the 2^−ΔΔCt^ method was used to determine the fold changes of the candidate genes (Qian et al., 2020).

**Table 1:** PCR primers for qRT-PCR.

Genes	Primer direction	Sequence (5′-3′)
*lasR*	F	GCTGGAACGCTCAAGTGG
	R	GGGTAGTTGCCGACGATG
*rhlI*	F	GTCGGTCTGGGAGCTTTCG
	R	CTCGCCCTTGACCTTCTGC
*rhlR*	F	CTCCTCGGAAATGGTGGTCT
	R	GCTTCTGGGTCAGCAACTCG
*pqsD*	F	GTCTGGGCAACATGGCTTCG
	R	ATGGCCGGTTCACCTCCTC
*pqsE*	F	AGCTGGGCGCTGGTTGAA
	R	GGTCGTAGTGCTTGTGGGTGAT
*atpF*	F	GTCCGTTGCGTTCTTCATCTTTGTG
	R	CAGGCCGTCAGCGATCTTCTTC
*sucD*	F	GGTGGTTCCGCCGAAGAAGAAG
	R	GCAGTCACACCAGCGATGTAGG
*phzS*	F	AGGACGTGCTGCCGTTCTTC
	R	GCGGATCGCGGTCTACCAT
*flgB*	F	CGCCGAGCAGAAGGACAA
	R	GGTGAAGGACGCCTGGAAGT
*mexF*	F	GTTCGCCAAGGACAAGCAGGAG
	R	CGGCACCACACCCATGATGAAG
*rpsL*	F	GCAACTATCAACCAGCTGGTG
	R	GCTGTGCTCTTGCAGGTTGTG

### 2.11. Docking analysis

The crystal structure file of the complex was downloaded from the protein crystal structure database (Protein Data Bank, http://www.rcsb.org/pdb/) as the basis for molecular docking. Molecular docking was performed using the Surflex-Dock module in SYBYL-X 2.0 (Chen et al., 2022; Qi et al., 2022a,b). During pretreatment, the ligands and water molecules in the complex 2OZ7 were deleted, and only the structural part of the protein was retained. Hydrogen atoms were added to the amino acid residues of the protein to repair the missing amino acid residues and side chains. The AMBER7 FF99 force field was used to minimize the protein and the gradient threshold was set to 0.01 kcal/(mol*A). The cutoff for non-bonded interactions was set to 8.00 and the dielectric constant was set to 1.00. After preprocessing the protein, the active pocket was determined based on the specific binding mode of the original ligand in the crystal structure 2OZ7, and the docking active pocket parameters were set. The threshold value (Threshold) was set to 0.50 and the expansion coefficient (Bloat) was set to 0. The ligand small molecules that have been constructed and optimized were selected to be docked with the protein, respectively. The specific binding mode between the protein and the ligand small molecule was obtained. Finally, the docking results were evaluated by the obtained scoring function.

### 2.12. Pathogenicity inhibition assay in lettuce

Overnight cultures of *P. aeruginosa* PAO1 were 0.1% diluted into LB broth with 1 mg/ml of thymoquinone, 2 mg/ml of thymoquinone, 1 mg/ml of nisin, 1 mg/ml of nisin+1 mg/ml of thymoquinone, 1 mg/ml of nisin+2 mg/ml of thymoquinone, and cultivated at 37°C for 17 h. DMSO and carvacrol (1 mg/ml) were selected as the negative and positive control, respectively. Lettuce was inoculated into 10 μl of the treated cultures and then cultivated at 37°C for 48 h. Each treatment was carried out three times independently. After cultivation, the rotten areas were recorded and weighed as described previously (Zhou et al., 2021).

### 2.13. Statistical analysis

All data were expressed as means ± standard deviation with three biological replicates. SPSS 18.0 software (SPSS, Inc., Chicago, IL, USA) was used for one-way ANOVA analysis at *p* ≤ 0.05. Origin 8.6 software (OriginLab, Northampton, MA, USA) was selected for graph processing.

## 3. Results

### 3.1. MIC and growth profile

**Figure 1:**
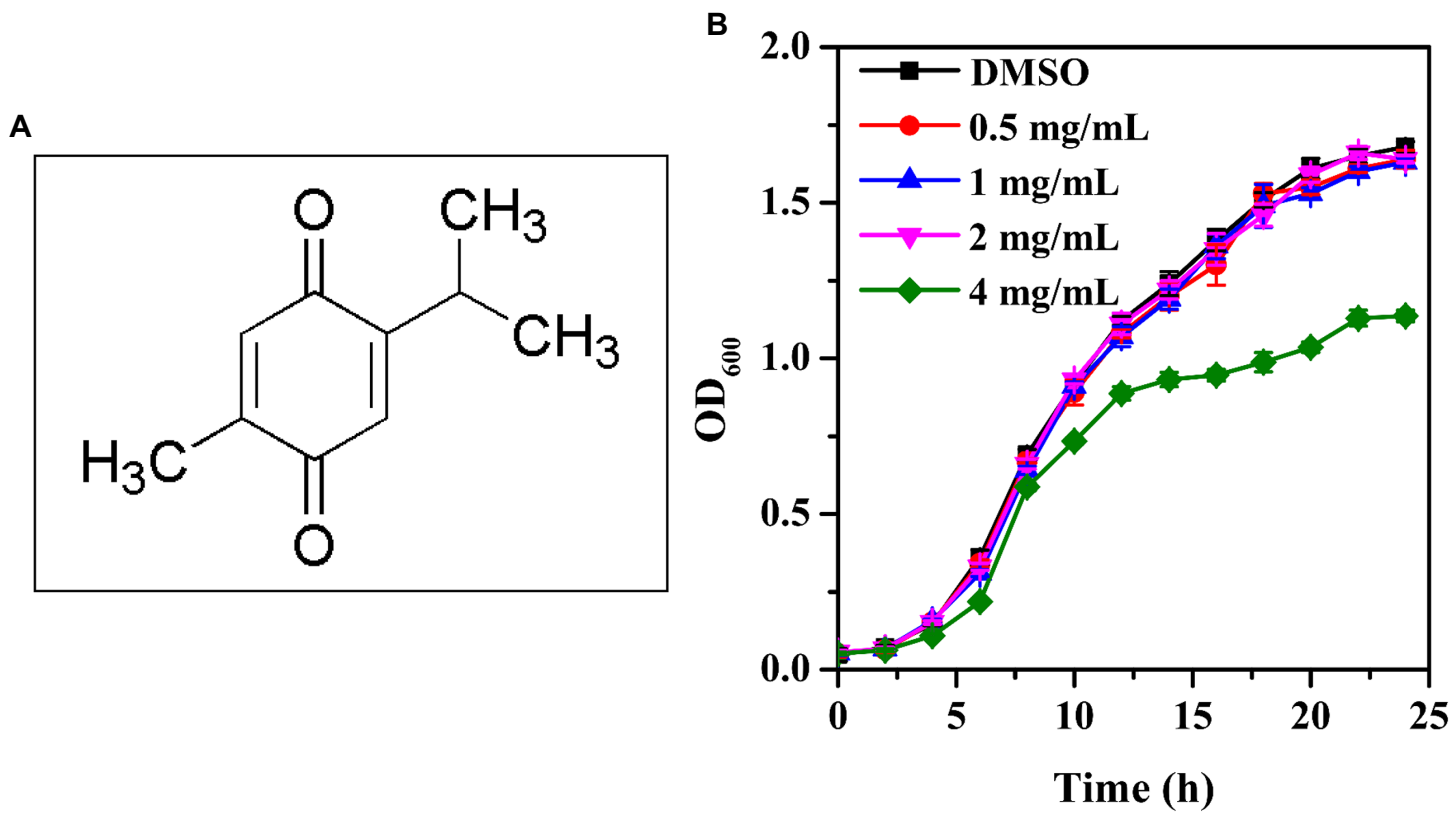
Chemical structure of thymoquinone **(A)** and growth profile of *P. aeruginosa* PAO1 following exposure to 0.5, 1, 2, and 4 mg/ml of thymoquinone for 24 h **(B)**. DMSO served as the negative control. Error bars represent standard deviations of three measurements.

The chemical structure of thymoquinone was shown in Figure 1A. The MICs of thymoquinone and nisin against *P. aeruginosa* PAO1 were both >8 mg/ml, respectively. The growth profile indicated that at sub-MIC concentrations ranging from 0.5 to 2 mg/ml, thymoquinone had no inhibitory impact on cell growth compared with the DMSO control (Figure 1B). However, treatment with thymoquinone at 4 mg/ml showed inhibitory effect on planktonic cell growth (Figure 1B).

### 3.2. Analysis of autoinducers production

**Figure 2:**
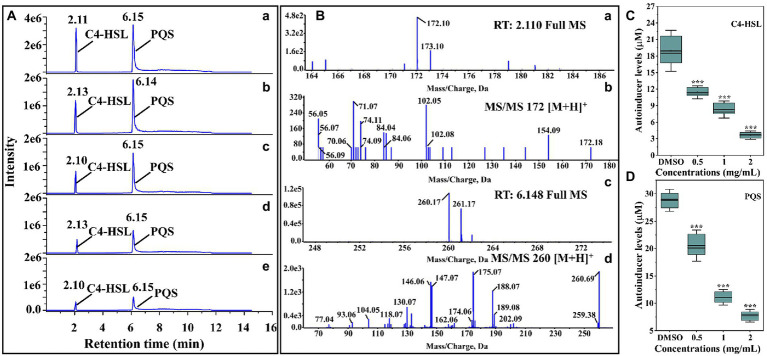
Identification and quantification of C4-HSL and PQS by LC–MS/MS chromatograms. **(A)** HPLC chromatograms of C4-HSL and PQS secreted by *P. aeruginosa* PAO1 supplemented with **(b)** DMSO, **(c)** 0.5, **(d)** 1, and **(e)** 2 mg/ml of thymoquinone. **(a)** Standard chemicals of C4-HSL and PQS. **(B)** MS and MS/MS spectra of C4-HSL and PQS, respectively: **(a)** full MS spectra of C4-HSL, **(b)** MS^2^ spectra of C4-HSL, **(c)** full MS of PQS, **(d)** MS^2^ spectra of PQS. **(C)** Quantitative analysis of C4-HSL treated with 0.5 to 2 μl/ml of thymoquinone. **(D)** Quantitative analysis of PQS treated with 0.5 to 2 μl/ml of thymoquinone.

The anti-QS activity of thymoquinone was firstly evaluated by measuring the autoinducers levels produced by *P. aeruginosa* PAO1. The HPLC chromatograms of the standard autoinducers presented retention time of 2.11 and 6.15 min corresponding to C4-HSL and PQS, respectively (Figure 2Aa). The MS spectra indicated that the [M + H]^+^ ions of 172.10 (Figure 2Ba) and 260.17 (Figure 2Bc) was corresponding to C4-HSL and PQS, respectively, and their presence was further validated by MS/MS spectra (Figures 2Bb,d). The quantification analysis indicated that the secretion of C4-HSL and PQS was significantly reduced after treatment with thymoquinone. Thymoquinone treatment at 0.5, 1, and 2 mg/ml resulted in the reduction of C4-HSL from 18.95 μM to 11.40, 8.29, and 3.65 μM, respectively (Figure 2C). A significant inhibition was also observed in PQS (Figure 2D). These data implied that thymoquinone may possess potent anti-QS potential against *P. aeruginosa*.

### 3.3. Inhibition on virulence factors

**Figure 3:**
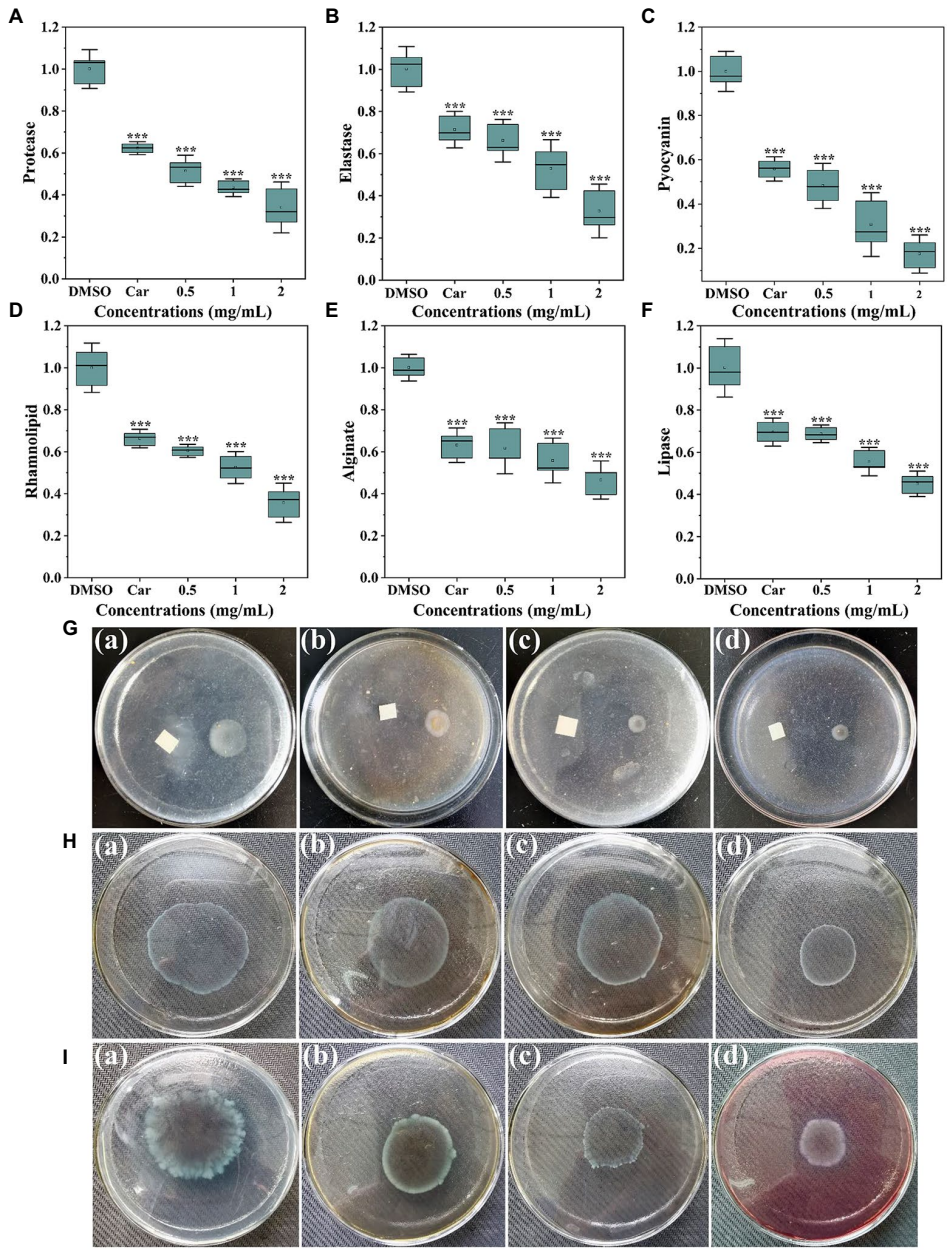
Inhibitory effects of thymoquinone on virulence factors production. **(A)** Protease activity. **(B)** Elastase activity. **(C)** Pyocyanin levels. **(D)** Rhamnolipid levels. **(E)** Alginate levels. **(F)** Lipase activity. Carvacrol (Car) and DMSO served as the positive and negative control, respectively. **(G-I)** represented chemotaxis, swimming motility, and swarming motility, respectively, treated with **(a)** DMSO, **(b)** 1 mg/ml of carvacrol, **(c)** 1 mg/ml of thymoquinone, and **(d)** 2 mg/ml of thymoquinone. Statistical differences were determined by ANOVA followed by the Tukey–Kramer test. (∗∗∗)*p* < 0.001 versus the DMSO control.

As shown in Figure 3A, protease activity was significantly inhibited with thymoquinone treatment. Exposure to thymoquinone at 2 mg/ml lead to a 66% reduction of protease compared to the DMSO control. This inhibitory effect was more effective than carvacrol, whose supplement lead to a 38% repression on protease activity. The impact of thymoquinone on elastase was presented in Figure 3B. Thymoquinone exposure (0.5–2 mg/ml) resulted in the reduction of elastase by approximately 34, 47, and 67%, respectively, and this reduced effect was dose-dependent. However, treatment with carvacrol at 1 mg/ml just resulted in a 29% decrease in elastase activity.

Pyocyanin is a characteristic virulence factor secreted by *P. aerugnosa* PAO1. Figure 3C indicated a markedly reduced level of pyocyanin treated with thymoquinone. At 0.5 mg/ml, about 52% decrease of pyocyanin was observed. At 2 mg/ml, thymoquinone reduced pyocyanin production by more than 80% compared to 44% by carvacrol. Rhamnolipid, another virulence factor produced by *P. aerugnosa* PAO1, was also markedly inhibited (Figure 3D). The inhibition of rhamnolipid was dose-dependent, with thymoquinone exposure (0.5–2 mg/ml) resulted in a decrease in rhamnolipid by about 40, 48, and 64%, respectively, compared to the control group.

As alginate plays vital roles in *P. aeruginosa* biofilm development and architecture (Stapper et al., 2004), the impact of thymoquinone on alginate production was investigated. Results indicated that alginate production was markedly decreased with increased administration of thymoquinone (Figure 3E). More than 50% decrease was detected after exposure to thymoquinone at 2 mg/ml. Similarly, thymoquinone administration also caused a suppressed effect on lipase activity (Figure 3F).

Thymoquinone was determined for its suppressed potential against chemotaxis, swimming and swarming motilities. For the DMSO-treated group, the chemotaxis diameter was approximately 1.9 ± 0.2 cm (Figure 3Ga). However, the chemotaxis diameter was decreased to 0.7 ± 0.3 cm (Figure 3Gc) and 0.4 ± 0.1 cm (Figure 3Gd), respectively, after exposure to thymoquinone at 1 and 2 mg/ml. The suppressed potential of thymoquinone was more effective than that of carvacrol (Figure 3Gb). Thymoquinone also exhibited concentration-dependent inhibition on swimming motility (Figure 3H). The swimming diameter was notably reduced from 4.6 ± 0.3 cm (Figure 3Ha) to 3.6 ± 0.3 cm (Figure 3Hc) and 2.3 ± 0.2 cm (Figure 3Hd), respectively, after treatment with thymoquinone at 1 and 2 mg/ml. A similarly notable inhibition was observed in swarming motility after treatment with thymoquinone (Figure 3I). The suppressed potential of thymoquinone was superior to that of carvacrol (Figure 3Ib).

### 3.4. Inhibition of biofilm formation on microplates

**Figure 4:**
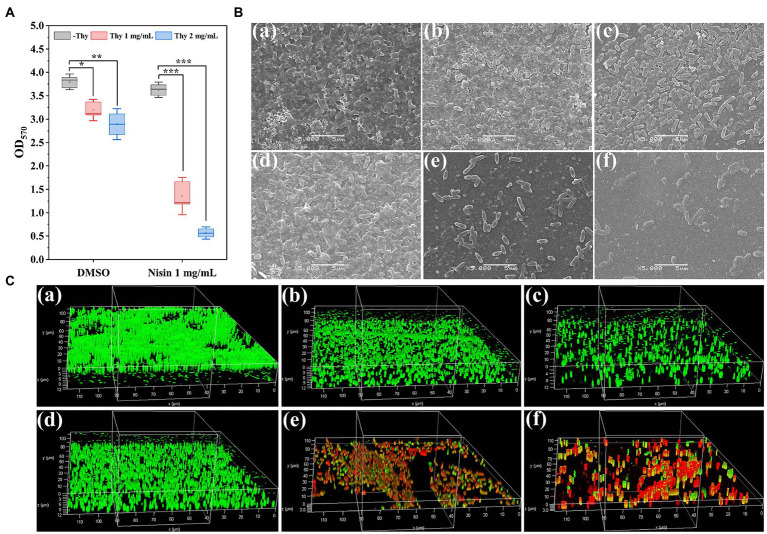
Inhibitory effect of thymoquinone and nisin on biofilm formation of *P. aeruginosa* PAO1 on microplates. **(A)** Quantification of biofilms using crystal violet staining method. **(B,C)** represented the SEM and CLSM images of biofilms, respectively, treated with **(a)** DMSO, **(b)** 1 mg/ml of thymoquinone, **(c)** 2 mg/ml of thymoquinone, **(d)** 1 mg/ml of nisin, **(e)** 1 mg/ml of nisin +1 mg/ml of thymoquinone, **(f)** 1 mg/ml of nisin +2 mg/ml of thymoquinone, respectively. Statistical differences were determined by ANOVA followed by the Tukey–Kramer test. (∗∗∗)*p* < 0.001 versus the DMSO control.

The synergistically inhibitory impact of thymoquinone and nisin on *P. aeruginosa* PAO1 biofilm formation was shown in Figure 4. Results indicated that nisin treatment individually had no significant inhibition on biofilm formation of *P. aeruginosa* PAO1 (Figure 4A). Exposure to thymoquinone at 1 and 2 mg/ml reduced biofilms by 16 and 24%, respectively. However, when treatment with thymoquinone in combination with nisin, a notably enhanced inhibitory effect was observed (Figure 4A). Treatment with thymoquinone (1 and 2 mg/ml) in combination with 1 mg/ml of nisin reduced biofilms by approximately 64 and 85%, respectively (Figure 4A). The result indicated that the amalgamation of thymoquinone with nisin was more effective against biofilm formation than agent used alone. This result was in accordance with the synergistic effect of hordenine and aminoglycoside antibiotics on *P. aeruginosa* PAO1 biofilms (Zhou et al., 2018a,b).

In order to visualize the synergistically inhibitory effect of thymoquinone and nisin on biofilms, samples were observed under SEM and CLSM (Figures 4B,C). The DMSO-treated group showed a dense and net-like structure attached with exopolysaccharides (Figure 4Ba), whereas thymoquinone exposure showed a scatter appearance (Figures 4Bb,c). When treatment with thymoquinone in combination with nisin, biofilms were markedly removed. The small remaining cell clusters were scatter and their integrity and the coated exopolysaccharides were significantly decreased (Figures 4Be,f). In addition, the CLSM images also showed a remarkable reduction of biofilm biomass exposed to thymoquinone and nisin (Figure 4C). When treatment with 1 mg/ml of nisin and 2 mg/ml of thymoquinone, biofilm thickness was decreased from 12.31 ± 2.16 μm (Figure 4Ca) to 3.08 ± 0.34 μm (Figure 4Cf), and higher mortality of bacterial cells was present as evidenced by the dominant red fluorescence.

### 3.5. Inhibition of biofilm formation on lettuce leaves

**Figure 5:**
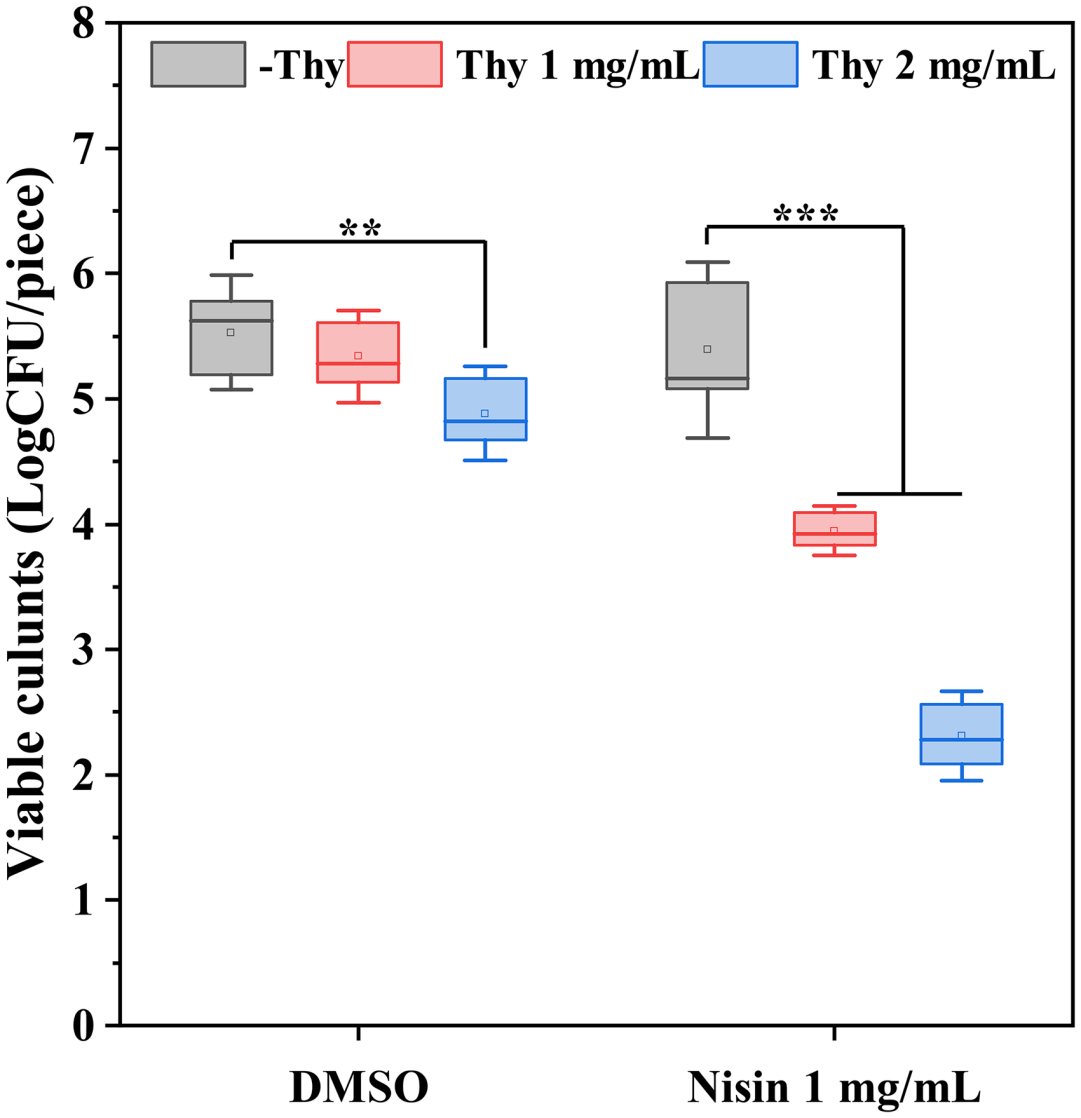
Effect of thymoquinone and nisin on biofilm formation of *P. aeruginosa* PAO1 on lettuce. Statistical differences were determined by ANOVA followed by the Tukey–Kramer test. (∗∗∗)*p* < 0.001 versus the DMSO control.

The synergistically inhibitory impact of thymoquinone and nisin on *P. aeruginosa* PAO1 biofilm formation on lettuce leaves was shown in Figure 5. Results indicated that nisin treatment individually had no significant inhibition on biofilm formation. Exposure to thymoquinone at 2 mg/ml reduced viable biofilm cells by 12%. However, when treatment with thymoquinone in combination with nisin, the inhibitory effect was notably enhanced. Treatment with nisin in combination with 1 and 2 mg/ml of thymoquinone reduced viable biofilm cells by 29 and 58%, respectively.

### 3.6. Identification of DEGs

**Figure 6:**
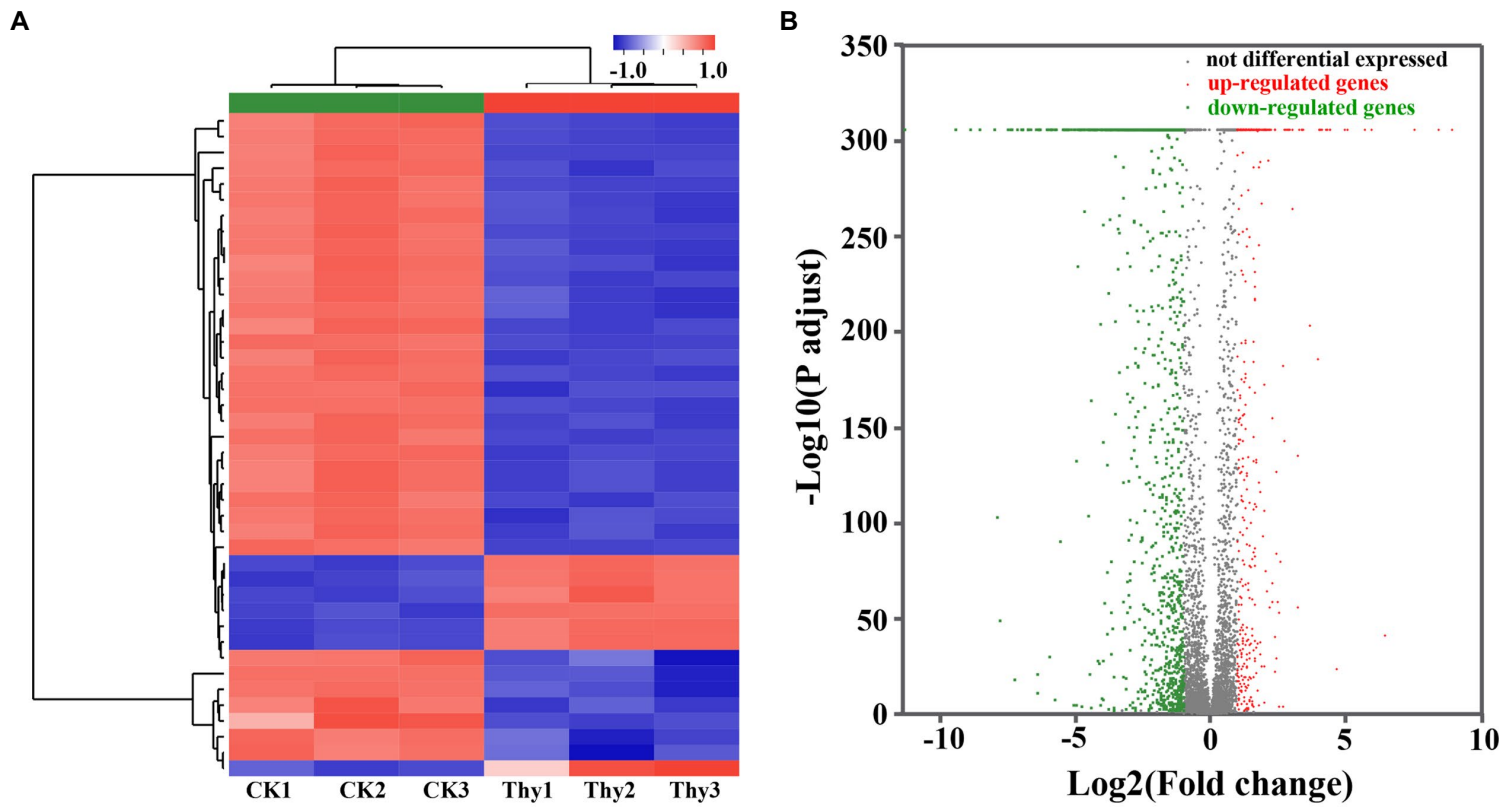
Heat map **(A)** and volcano plots **(B)** of DEGs in *P. aeruginosa* PAO1 after thymoquinone treatment. For heat map, each row represents the expression pattern of a single gene and each column corresponds to a single sample. The expression levels are represented by colored tags, with red representing the highest levels of expression and blue representing the lowest levels of expression. For volcano plots, the red dots represent up-regulated DEGs, the green dots represent down-regulated DEGs and the gray dots represent genes with no significant changes in expression.

A total of 28.41 Gb clean data were obtained after filtering through the raw reads. The observed Q30 for the RNA sequenced samples was ranged from 98.73 to 99.44%. The data from heat map (Figure 6A), volcanic map (Figure 6B), and log_2_ (ratio) presented the differentially expressed genes (DEGs) compared between DMSO and thymoquonone-treated groups. A total of 1,542 DEGs were screened, among which 563 genes were notably up-regulated, and 979 genes were down-regulated.

### 3.7. GO analysis

**Figure 7:**
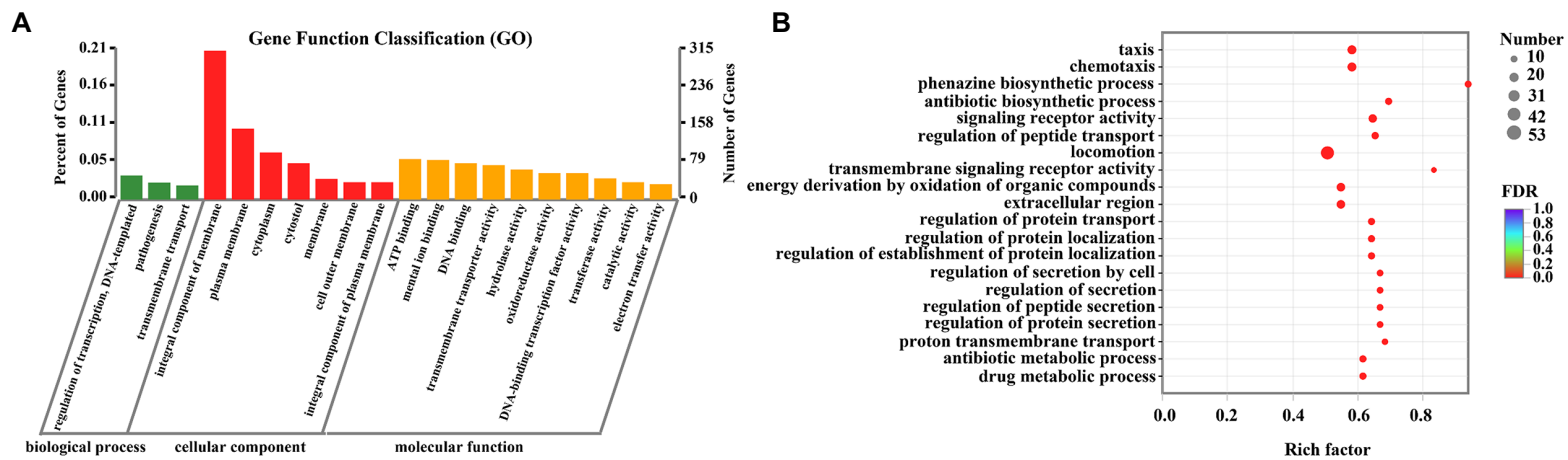
DEGs between the two sample groups were analyzed by GO. **(A)** Histogram of GO annotation of DEGs, including biological process, cellular component and molecular function. **(B)** GO enrichment analysis of DEGs.

GO functional enrichment analysis was presented in Figure 7. All DEGs were divided into three categories: biological process, cellular component, and molecular function, consisting of 3, 7, and 10 subcategories, respectively (Figure 7A). Integral component of membrane, plasma membrane, and cytoplasm were the main modules of DEGs distribution. For biological process, the most vital ones were regulation of transcription and pathogenesis. While for molecular function, ATP binding, mental ion binding, and DNA binding were the most representative DEGs. Figure 7B and Supplementary Table S1 presented the specific DEGs characteristics in each of the three modules. Results indicated that 15 DEGs were observed in phenazine biosynthesis, 25 in chemotaxis, 18 in type III secretion system, 36 in pathogenesis, 67 in motility, 171 in transmembrane transporter, and 63 in ATP biosynthetic process. The data from the GO enrichment indicated that thymoquinone treatment notably affected virulence factors synthesis, pathogenicity, infection ability, and energy metabolism of *P. aeruginosa* PAO1.

### 3.8. KEGG pathway analysis

**Figure 8:**
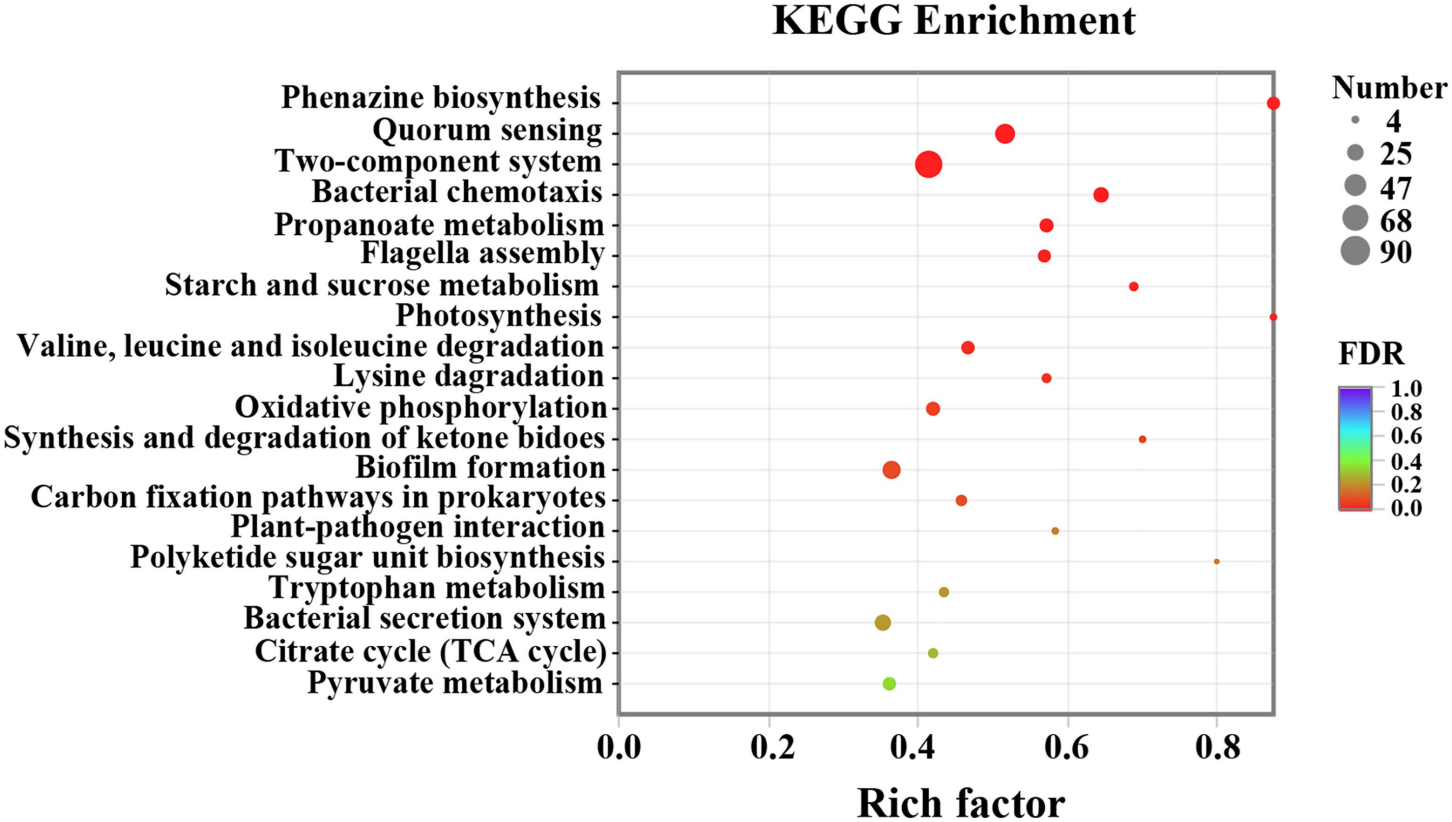
KEGG enrichment analysis of *P. aeruginosa* PAO1 after thymoquinone treatment.

To further investigate the biological process and functional pathways in which DEGs involved, KEGG enrichment was presented. The data indicated that 507 DEGs were annotated in the KEGG data library and enriched 165 categories. The first 20 variable pathways were used to screen out important genes in the KEGG enrichment pathway (Figure 8). Based on this result, 36 genes were selected as vital genes correlated with the measured phenotypic data of phenazine synthesis, QS, virulence factors, and drug resistance (Table 2).

**Table 2:** Some selected DEGs related to virulence and drug resistance.

Gene category	Log_2_ FC	Name	Gene description
Phenazine biosynthesis			
PA4210	−11.32	*phzA1*	phenazine biosynthesis
PA1899	−5.43	*phzA2*	phenazine biosynthesis
PA4211	−8.89	*phzB1*	phenazine biosynthesis
PA1900	−7.38	*phzB2*	phenazine biosynthesis
PA4213	−6.92	*phzD1*	phenazine biosynthesis
PA1902	−6.92	*phzD2*	phenazine biosynthesis
PA4214	−6.72	*phzE1*	phenazine biosynthesis
PA1903	−6.72	*phzE2*	phenazine biosynthesis
PA4215	−9.40	*phzF1*	phenazine biosynthesis
PA1904	−6.32	*phzF2*	phenazine biosynthesis
PA1905	−6.42	*phzG2*	phenazine biosynthesis
PA4209	−5.39	*phzM*	adenosylmethionine-dependent methyltransferase
PA4217	−5.89	*phzS*	flavin-comprising hydroxylase
Quorum sensing			
PA1430	−1.44	*lasR*	transcriptional regulator LasR
PA3476	−3.66	*rhlI*	acyl-homoserine-lactone synthase
PA3477	−3.29	*rhlR*	transcriptional regulator RhlR
PA0996	−7.12	*pqsA*	anthranilate--CoA ligase
PA0997	−6.31	*pqsB*	hypothetical protein
PA0998	−5.98	*pqsC*	hypothetical protein
PA0999	−4.79	*pqsD*	3-oxoacyl-ACP synthase
PA1000	−4.87	*pqsE*	thioesterase PqsE
PA2587	−1.40	*pqsH*	quinolone synthase
Virulence factors			
PA3479	−5.73	*rhlA*	rhamnosyltransferase subunit A
PA3478	−5.32	*rhlB*	rhamnosyltransferase subunit B
PA1130	−3.04	*rhlC*	rhamnosyltransferase
PA1871	−4.81	*lasA*	protease LasA
PA3724	−5.38	*lasB*	elastase LasB
PA2570	−5.88	*lecA*	PA-I galactophilic lectin
PA1077	−3.14	*flgB*	flagellar biosynthesis protein FlgB
PA1078	−3.02	*flgC*	flagellar biosynthesis protein FlgC
PA5261	−1.44	*algR*	alginate biosynthesis protein AlgR
PA3349	−1.78	*PA3349*	chemotaxis protein
Drug resistance			
PA2493	−2.41	*mexE*	resistance efflux protein
PA2494	−2.67	*mexF*	resistance efflux protein
PA2495	−1.67	*oprN*	resistance efflux transporter
Oxidative stress			
PA2147	−4.45	*katE*	catalase HPII
PA2185	−2.07	*katN*	non-heme catalase KatN
PA2826	−1.49	*PA2826*	glutathione peroxidase

#### 3.8.1 Phenazine biosynthesis

Phenazine compounds are important virulence factors secreted by *P. aeruginosa* APO1. Among which, pyocyanin is the representative and is essential for biofilm formation and infection process (Wu et al., 2016). Studies have proven that *phz1* and *phz2* operons play central roles in the biosynthesis of pyocyanin (Sultan et al., 2021). As indicated in Table 2, the phenazine biosynthesis genes *phzA1*, *phzA2*, *phzB1*, *phzB2*, *phzD1*, *phzD2*, *phzE1*, *phzE2*, *phzF1*, *phzF2*, *phzG2*, adenosylmethionine-dependent methyltransferase gene *phzM*, and flavin-comprising hydroxylase gene *phzS* were significantly down-regulated after thymoquinone treatment. This result was in accordance with the reduced pyocyanin levels.

#### 3.8.2 QS systems

*P. aeruginosa* possesses three QS systems, *las*, *rhl*, and *pqs*, which mediate the expressions of thousands of genes. And many of these genes are related to virulence factors, preservative resistance, and pathogenesis (Sandoz et al., 2007). Therefore, blocking QS is an effective approach for attenuating the virulence and resistance of *P. aeruginosa*. Table 2 showed that the *las* system (*lasR*), *rhl* system (*rhlI* and *rhlR*), and *pqs* system (*pqsA*, *pqsB*, *pqsC*, *pqsD*, *pqsE*, and *pqsH*) were significantly inhibited after thymoquinone treatment. The broken QS would inevitably lead to the decreased pathogenicity and drug resistance of *P. aeruginosa*.

#### 3.8.3 Virulence factors

As *P. aeruginosa* uses QS to coordinate the synthesis of many virulence factors including protease, elastase, rhamnolipid, alginate, lipase, and so on (Wang et al., 2019), the efficiency of thymoquinone on expressions of genes involved in virulence was determined. As indicated in Table 2, rhamnolipid synthesis genes (*rhlA*, *rhlB*, and *rhlC*), protease synthesis gene (*lasA*), elastase synthesis gene (*lasB*), lectin synthesis gene (*lecA*), alginate synthesis gene (*algR*), flagella synthesis genes (*flgB* and *flgC*), and chemotaxis gene (PA3349) were significantly inhibited with thymoquinone exposure. This result was in accordance with the disrupted QS and the suppressed phenotypes of virulence factors.

#### 3.8.4 Drug resistance

Studies have proven that the MexEF-OprN multidrug efflux pump controls drug resistance and virulence in *P. aeruginosa* (Aendekerk et al., 2005). In this study, the multidrug efflux pump genes *MexE*, *MexF*, and *oprN* were notably down-regulated in the presence of thymoquinone. This result was correlated well with the enhanced susceptibility of biofilms to the combination of nisin and thymoquinone.

#### 3.8.5 Oxidative stress

The expressions of genes related to detoxifying enzymes were also sorted out. As presented in Table 2, the expressions of *katE*, *katN*, and *PA2826* encoding catalase and glutathione peroxidase, respectively, were significantly down-regulated after exposure to thymoquinone.

### 3.9. Intracellular metabolites

^1^H NMR-based metabonomics approach was employed to interrogate the metabolites related to protein synthesis, antioxidation, membrane composition, and energy metabolism. As presented in Table 3, a notable reduction in isoleucine, leucine, glutathione, betaine, ethanolamine and fumarate, and a prominent increase in glutamate, succinate, and glycine were identified in the thymoquinone-treated group. The detailed assignments, chemical shifts, fold changes and significance marks were listed in Table 3.

**Table 3:** The assigned metabolites involved in protein synthesis, antioxidation, membrane composition, and energy metabolism.

No.	Metabolites	Assignments	Chemical shift^a^ (ppm)	Fold change^b^	*p* value^c^
1	Isoleucine	*δ*-CH_3_, *β*-CH_3_	0.94 (t), 1.01 (d)	−0.71	**
2	Leucine	*δ*-CH_3_, CH_2_	0.96 (t), 1.71 (m)	−0.36	*
3	Valine	CH_3_, CH_3_	0.99 (d), 1.04 (d)	0.11	
4	Glutamate	*β*-CH_2_, *α*-CH_2_, *N*-CH	2.07(m),2.35(m)	0.83	***
5	Glutathione	CH_2_	2.18(m),2.52(m)	−0.65	***
6	Succinate	CH	2.4(s)	1.12	***
7	Betaine	*α*-CH_2_	3.27(s)	−0.49	**
8	Ethanolamine	*N*-CH_2_, CH_2_	3.15(t), 3.83(t)	−0.55	***
9	Glycine	*α*-CH_2_	3.595(s)	0.51	**
10	Fumarate	CH	6.525(s)	−0.86	***

a Multiplicity: (s) singlet, (d) doublet, (t) triplet, (m) multiplets.

b Color-coded according to the log_2_(fold): red and blue represent the increased and decreased metabolites, respectively, in thymoquinone-treated group.

c *p* values were calculated based on a parametric Student t test or a nonparametric Mann–Whitney test and were corrected by the BH (Benjamini-Hochberg) methods.

* *p* < 0.05 (values with asterisk symbol denoted extent of significance).

** *p* < 0.01 (values with asterisk symbol denoted extent of significance).

*** *p* < 0.001 (values with asterisk symbol denoted extent of significance).

### 3.10. Oxidative stress and membrane permeability

**Figure 9:**
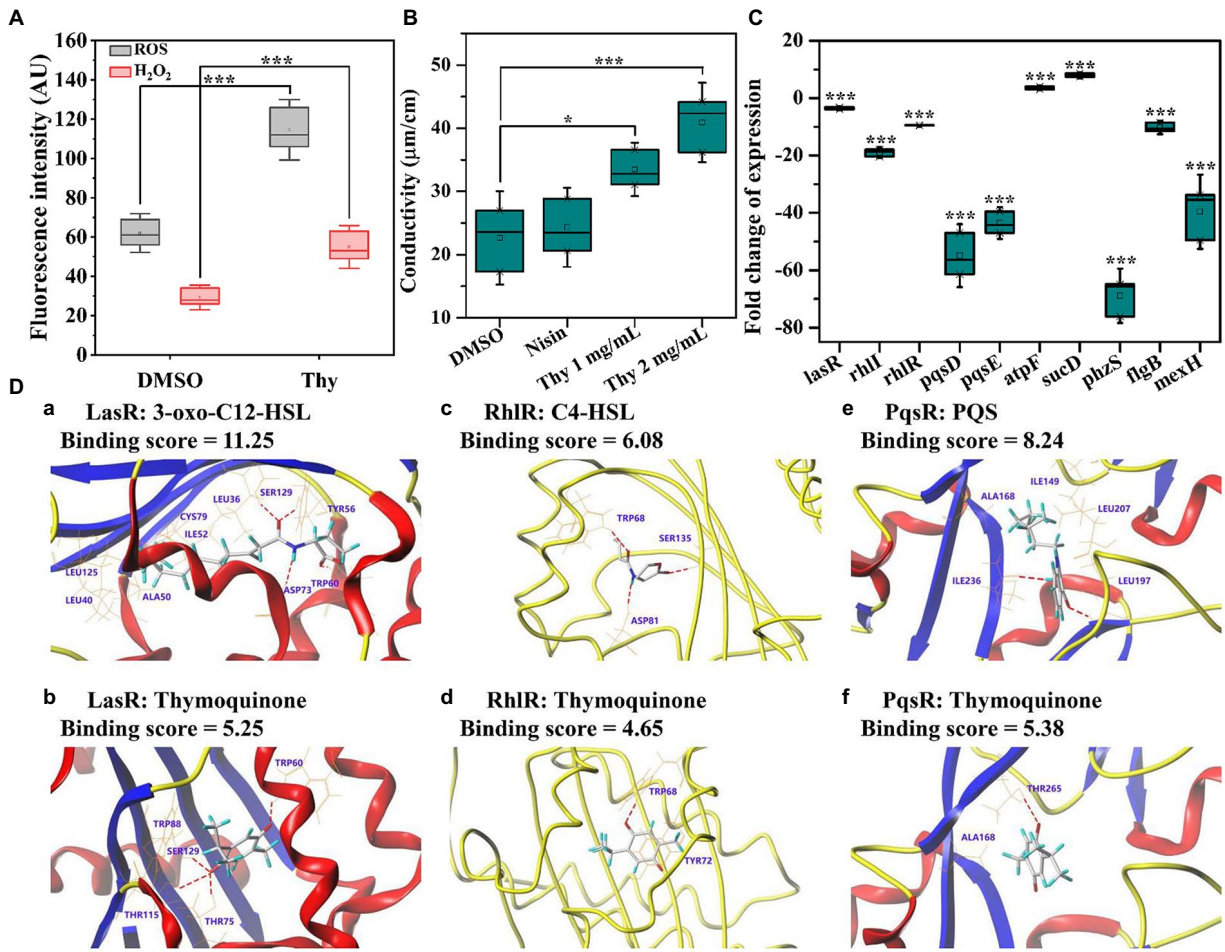
Effects of thymoquinone on ROS production, cell membrane permeability, gene expression, and the docking sumulation. **(A)** ROS and H_2_O_2_ production. **(B)** Membrane permeability. **(C)** Expressions of genes involved in QS, virulence, energy metabolism, and drug resistance. **(D)** A 3D schematic of receptor-ligand interactions of LasR **(a,b)**, RhlR **(c,d)**, and PqsR **(e,f)** with 3-oxo-C12-HSL **(a)**, C4-HSL **(c)**, PQS **(e)**, and thymoquinone **(b,d,f)**, respectively. Statistical differences were determined by ANOVA followed by the Tukey–Kramer test. (∗∗∗)*p* < 0.001 versus the DMSO control.

The impact of thymoquinone on ROS and H_2_O_2_ release was presented in Figure 9A. Exposure to thymoquinone at 2 mg/ml resulted in a notably enhanced production of ROS and H_2_O_2_. The result implied that *P. aeruginosa* PAO1 suffered from serious oxidative damage after thymoquinone administration. Membrane permeability assay showed that no obvious change was detected in membrane permeability of the DMSO and nisin-treated samples after 24-h cultivation (Figure 9B). However, after thymoquinone treatment, the membrane permeability was significantly enhanced (Figure 9B).

### 3.11. RT-qPCR confirmation

A total of 10 DEGs enriched into KEGG pathway were selected and their relative expressions were validated by RT-qPCR. As shown in Figure 9C, the expressions of *lasR*, *rhlI*, *rhlR*, *pqsD*, *pqsE*, *phzS*, *flgB*, and *mexF* were down-regulated by 3.6, 19.0, 9.5, 54.9, 43.6, 68.9, 10.3, and 39.6 folds, respectively, while *atpF* and *sucD* related to energy metabolism were up-regulated by 3.6 and 7.9 folds, respectively, after thymoquinone exposure. These results were in accordance with the transcriptome data and confirmed the reliability of transcriptome analysis.

### 3.12. Docking analysis

The binding affinity and potential binding sites of thymoquinone to LasR, RhlR, and PqsR were investigated by docking analysis (Figure 9D). The carbonyl groups of thymoquinone formed H-binding interactions with TRP60, THR75, THR115, and SER129, respectively (Figure 9Db). However, the carbonyl groups and amino residue of 3-oxo-C12-HSL formed H-binding interactions with TRP60, SER129, TYR56, and ASP73, respectively (Figure 9Da). The binding score of thymoquinone with LasR was lower than that of 3-oxo-C12-HSL (Figures 9Da,b). This indicated that thymoquinone has lower binding affinity with LasR. The binding score of thymoquinone with RhlR was 4.65, which was lower than that of C4-HSL (Figures 9Dc,d). The carbonyl group of thymoquinone was bound to RhlR with TRP68 (Figure 9Dd), while the carbonyl groups and amide group of C4-HSL exhibited H-bonding interactions with TRP68, SER135, and ASP81, respectively (Figure 9Dc). In addition, the carbonyl group of thymoquinone showed H-interaction with THR265 (Figure 9Df). The hydroxyl group and carbonyl group of PQS formed H-interactions with ILE236 and LEU197, respectively (Figure 9De). The binding score indicated that PQS has stronger binding affinity to PqsR than that of thymoquinone.

### 3.13. Lettuce infection assay

**Figure 10:**
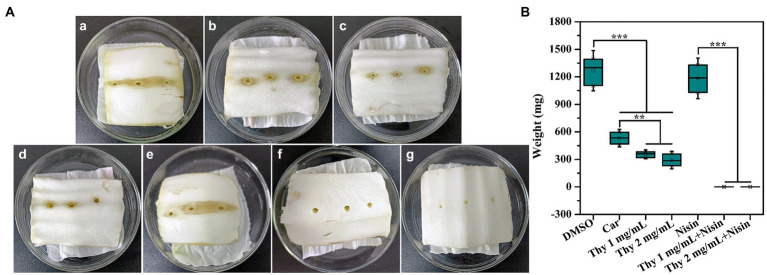
Effect of thymoquinone on lettuce infections. **(A)** Pictures of inoculation of *P. aeruginosa* PAO1 into lettuces treated with **(a)** DMSO, **(b)** 1 mg/ml of carvacrol, **(c)** 1 mg/ml of thymoquinone, **(d)** 2 mg/ml of thymoquinone, **(e)** 1 mg/ml of nisin, **(f)** 1 mg/ml of nisin +1 mg/ml of thymoquinone, and **(g)** 1 mg/ml of nisin +2 mg/ml of thymoquinone. DMSO and carvacrol were used as negative and positive control, respectively. **(B)** Quantification of soft-rot weight of lettuces treated with or without thymoquinone. (∗∗∗)*p* < 0.001 versus the DMSO control.

The efficiency of thymoquinone on vegetable plant infection was determined. As presented in Figure 10, thymoquinone administration at 1 and 2 mg/ml significantly reduced the infection of *P. aeruginosa* PAO1 on lettuce. The rotten area and weight of lettuce was notably reduced after thymoquinone treatment compared to the carvacrol and DMSO-treated samples. Most importantly, when treatment with thymoquinone in combination with 1 mg/ml of nisin, lettuce rot was almost completely inhibited. The result indicated that thymoquinone can serve as an effective agent in treating vegetable plants infection.

## 4. Discussion

Nisin, a 34 amino acid polycyclic peptide, has antimicrobial potential against a wide range of foodborne pathogens and is widely used as preservative (Field et al., 2016b). Studies have shown that nisin has potent anti-virulence and anti-biofilm activities against a large amount of Gram-positive bacteria (Field et al., 2016a,b). However, the anti-virulence activity of nisin against Gram-negative bacteria especially *P. aeruginosa* is weak. Therefore, it is urgent to enhance the anti-virulence activity of nisin and make it more widely used in food industry. An alternative option is the use of nisin in combination with other natural anti-virulence agents. Thymoquinone, a black seed-producing natural molecule, has traditionally been used as antioxidant and antimicrobial agent (Khader et al., 2009; Darakhshan et al., 2015). Previous research has shown that thymoquinone possesses anti-biofilm activity against the food-borne pathogen *P. aeruginosa* in combination with tetrazine-capped silver nanoparticles and tryptophan (Chakraborty et al., 2021). However, whether thymoquinone could enhance the inhibitory effect of nisin on *P. aeruginosa* biofilms has not been reported, and the potential anti-virulence mechanism of thymoquinone remained uncovered. In this study, thymoquinone was investigated for its ability to inhibit QS-mediated virulence factors and biofilm formation. The potential anti-virulence and anti-biofilm mechanisms of thymoquinone were determined using transcriptome, metabolome, and docking analysis.

Protease, elastase, pyocyanin, rhamnolipid, alginate, and lipase are extracellular virulence factors secreted by *P. aeruginosa*. These virulence factors are mediated by QS and play vital roles in infection, drug-resistance, pathogenicity, or food spoilage (Newman et al., 2017; Machado et al., 2020). Therefore, inhibition of virulence factors is an indicator of dysfunctional QS and also an effective approach for attenuating the pathogenicity of *P. aeruginosa*. The results indicated that the production of protease, elastase, pyocyanin, rhamnolipid, alginate, and lipase were all inhibited after exposure to thymoquinone. The results were correlated well with the broken QS and repressed expressions of genes involved in virulence. The approach for disrupting QS can be attributed to signal mimicry or signal degradation, which lead to the suppression of downstream virulence-related genes (Ni et al., 2009). In this study, docking analysis indicated that thymoquinone has lower binding affinity with LasR, RhlR, and PqsR. This result combined with the transcriptome data and autoinducers quantification revealed that thymoquinone functions as an anti-QS agent through repressing the transcriptional levels of QS-related genes rather than signal mimicry. The blocked QS would inevitably result in the inhibition of motilities since these physiological activities were all QS-mediated (Zhou et al., 2021). And this speculation was verified by the inhibited chemotaxis, swimming, and swarming assay.

Given the vital role of QS in biofilm formation, the synergistic effect of thymoquinone with nisin against *P. aeruginosa* PAO1 biofilm formation both on microplates and lettuce leaves was investigated. The result indicated that thymoquinone-treated biofilms were structurally changed. The sessile cells were flat and scattered, and the integrity was also destroyed. When exposure to thymoquinone in combination with nisin, the anti-biofilm efficiency was significantly enhanced. This enhanced effect may be induced, at least partially, by the altered structure and membrane permeability after thymoquinone exposure. As is known that rhamnolipid is a vital surfactant and plays significant roles in cells motility and biofilm architecture (Davey et al., 2003). Alginate, a well-known virulence factor produced by *P. aeruginosa* PAO1, is also essential for biofilm development and architecture (Ryder et al., 2007). The reduced production of rhamnolipid and alginate would inevitably result in the altered architecture of biofilms, which was evidenced by the SEM analysis. In addition, the CLSM analysis indicated that the combination of thymoquinone and nisin resulted in the entrance of antibiotics into biofilm cells and induced the killing of cells, as validated by the dominant red fluorescence. This result revealed that the permeability of cell membrane might be enhanced. It has been evidenced that the multidrug resistance of *P. aeruginosa* is induced by a synergy between a low-permeability outer membrane and the activated multidrug efflux systems including MexEF-OprN (Schweizer, 2003). In order to clarify the membrane permeability, metabolome technology was employed. As is known that ethanolamine is an important component of cellular membranes and plays vital roles in maintaining membrane permeability (Zhou et al., 2019b). The reduced ethanolamine level through ^1^H NMR-based metabonomics revealed that the membrane permeability was markedly altered (Table 3). The altered membrane permeability facilitated the entrance of nisin into the treated biofilms and enhanced the susceptibility of biofilms to nisin. In addition, transcriptome analysis (Table 2) and RT-qPCR (Figure 9B) assay indicated that the expressions of efflux pump genes *mexE*, *mexF*, and *oprN* were severely inhibited. Since MexEF-OprN system acts as important roles in exporting of antimicrobials (Li et al., 1998), interfering with its expression could be an effective strategy for reducing the drug resistance of *P. aeruginosa*. In this study, the repressed MexEF-OprN system and the enhanced membrane permeability resulted in decreased nisin resistance and facilitated the entrance of antibiotics into biofilm cells.

In addition, transcriptome data showed that the expressions of genes involved in catalase and glutathione peroxidase were significantly inhibited (Table 2). Since catalase and glutathione peroxidase are critical antioxidant enzymes for defending against oxidative stress, the inhibition of these two enzymes would inevitably result in the enhancement of oxidative damage. This speculation was supported by the increased levels of ROS and H_2_O_2_, and the changed glutamate and glutathione levels (Table 3). Glutathione peroxidase can catalyze the transformation of glutathione into oxidized glutathione and reduce toxic peroxides into non-toxic hydroxyl compounds, so as to protect the structure and function of cell membrane from oxide interference and damage (Weydert and Cullen, 2010). The decrease of glutathione peroxidase activity would lead to the increase of glutathione and glutamate. However, in this study, the glutathione level was significantly decreased after thymoquinone treatment. This decreased glutathione further revealed the enhanced oxidative injury. In order to defend against the enhanced oxidative injury, glutathione was over consumed (Zhou et al., 2019a). Isoleucine, leucine, and valine are branched chain amino acids (BCAAs) and play important roles in protein synthesis (Zhou et al., 2019b). The changed levels of BCAAs demonstrated a disorder of protein metabolism due to the broken QS and enhanced oxidative stress (Zhou et al., 2019a). In addition, the notable increase of succinate and decrease of fumarate indicated the disorder of energy metabolism as they are intermediates of the tricarboxylic acid (TCA) cycle. This speculation was also confirmed by the GO enrichment analysis and RT-qPCR data. As the most important energy supply for organisms, the disruption of TCA cycle would inevitably lead to the disorder of energy supply and ultimately attenuate the pathogenicity of *P. aeruginosa* PAO1 (Zhou et al., 2019a).

## 5. Conclusion

In this study, the anti-virulence activity and mechanism of quimoquinone against the foodborne pathogen *P. aeruginosa* were evaluated. Thymoquinone exposure significantly reduced the virulence factors and showed synergistically inhibitory effect on *P. aeruginosa* PAO1 biofilm formation both on microplates and lettuce leaves in combination with nisin. The anti-virulence mechanism can be attributed to the dysfunctional QS and repressed genes involved in virulence. The enhanced susceptibility of biofilms to nisin was mainly due to the improved membrane permeability and the repressed MexEF-OprN system. In addition, the antioxidant enzymes were inhibited and oxidative stress was intensified. The intensified oxidative stress disturbed energy metabolism, protein metabolism, and ultimately attenuated the pathogenicity of *P. aeruginosa* PAO1. These results provide new insight into the possible underlying anti-virulence mechanism of thymoquinone and deepen our understanding of the response of *P. aeruginosa* to thymoquinone.

## Data availability statement

The datasets presented in this study can be found in online repositories. The names of the repository/repositories and accession number(s) can be found in the article/Supplementary material.

## Author contributions

HC and J-WZ designed the experiments and analyzed and wrote the manuscript. HC, P-CJ, Y-HQ, S-JC, C-YW, Y-JY, and X-YZ performed the experiments. All authors contributed to the article and approved the submitted version.

## Funding

This work was supported by grants from the National Natural Science Foundation of China (32000091), General Projects of Natural Science Research in Universities of Jiangsu Province (20KJB180019), Jiangsu Youth Talent Promotion Project (TJ-2021-066), Henan Province Science and Technology Attack Plan Foundation (212102110241 and 222102310356), the Key Scientific Research Project of Higher Education of Henan Province (21A350006 and 22B350004), Natural Science Foundation of Jiangsu Province (BK20211051), Natural Science Foundation of Jiangsu Province (BK20210078), Natural Science Foundation of the Jiangsu Higher Education Institutions (22KJA240003), and Key Research and Development Plan of Xuzhou City (KC21296).

## Conflict of interest

The authors declare that the research was conducted in the absence of any commercial or financial relationships that could be construed as a potential conflict of interest.

## Publisher’s note

All claims expressed in this article are solely those of the authors and do not necessarily represent those of their affiliated organizations, or those of the publisher, the editors and the reviewers. Any product that may be evaluated in this article, or claim that may be made by its manufacturer, is not guaranteed or endorsed by the publisher.
