# Supplementary material for: Inactivation of Pseudomonas aeruginosa biofilms by thymoquinone in combination with nisin
Source: Front Microbiol. 2023 Jan 19;13:1029412. doi: 10.3389/fmicb.2022.1029412 (PMC9893119; doi:10.3389/fmicb.2022.1029412)
Supplement: Supplementary file 1 [file Table_1.docx]

Supplementary Material

**Inactivation of *Pseudomonas aeruginosa* biofilms on lettuce by thymoquinone in combination with nisin**

Hong Chen^a, #^, Peng-Cheng Ji^b, #^, Yue-Heng Qi^a^, Shi-Jin Chen^a^, Chang-Yao Wang^b^, Yu-Jie Yang^b^, Xin-Yu Zhao^b^, Jin-Wei Zhou^b*^

^a^Luoyang Key Laboratory of Organic Functional Molecules, College of Food and Drug, Luoyang Normal University, Luoyang, 471934, China

^b^School of Food and Biological Engineering, Xuzhou University of Technology, Xuzhou, 221018, China

^#^These authors contributed equally to this work.

**^*^Corresponding author:**

*E-mail address:* zhoujw86@126.com

Tel: 86-516-83105383. Fax: 86-516-83105383.

**Table S1.** The specific DEGs characteristics in each of the three modules biological process (BP), cellular component (CC), and molecular function (MF)

| GO ID | Function Description | Category | Gene Num | P value |
| --- | --- | --- | --- | --- |
| GO:0002047 | phenazine biosynthesis | BP | 15 | 0.00 |
| GO:0040011 | locomotion | BP | 53 | 0.00 |
| GO:0042330 | taxis | BP | 25 | 0.00 |
| GO:0006935 | chemotaxis | BP | 25 | 0.00 |
| GO:0017000 | antibiotic biosynthesis | BP | 16 | 0.00 |
| GO:0090087 | regulation of peptide transport | BP | 17 | 0.00 |
| GO:0015980 | energy derivation | BP | 23 | 0.00 |
| GO:0051223 | regulation of protein transport | BP | 16 | 0.00 |
| GO:0032880 | regulation of protein localization | BP | 16 | 0.00 |
| GO:0070201 | regulation of establishment of protein localization | BP | 16 | 0.00 |
| GO:1903530 | regulation of secretion by cell | BP | 14 | 0.00 |
| GO:0051046 | regulation of secretion | BP | 14 | 0.00 |
| GO:0002791 | regulation of peptide secretion | BP | 14 | 0.00 |
| GO:0050708 | regulation of protein secretion | BP | 14 | 0.00 |
| GO:1902600 | proton transmembrane transport | BP | 13 | 0.00 |
| GO:0016999 | antibiotic metabolic process | BP | 16 | 0.00 |
| GO:0017144 | drug metabolic process | BP | 16 | 0.00 |
| GO:0044419 | interspecies interaction between organisms | BP | 40 | 0.00 |
| GO:0006754 | ATP biosynthetic process | BP | 9 | 0.00 |
| GO:0044275 | cellular carbohydrate catabolic process | BP | 9 | 0.00 |
| GO:1904950 | negative regulation of protein localization | BP | 10 | 0.00 |
| GO:0051224 | negative regulation of protein transport | BP | 10 | 0.00 |
| GO:0098662 | inorganic cation transmembrane transport | BP | 23 | 0.00 |
| GO:0019471 | 4-hydroxyproline metabolic process | BP | 6 | 0.00 |
| GO:0019470 | 4-hydroxyproline catabolic process | BP | 6 | 0.00 |
| GO:0030254 | type III secretion system | BP | 18 | 0.00 |
| GO:0015985 | energy coupled proton transport | BP | 7 | 0.00 |
| GO:0015986 | ATP synthesis coupled proton transport | BP | 7 | 0.00 |
| GO:0051048 | negative regulation of secretion | BP | 9 | 0.00 |
| GO:0002792 | negative regulation of peptide secretion | BP | 9 | 0.00 |
| GO:0050709 | negative regulation of protein secretion | BP | 9 | 0.00 |
| GO:1903531 | negative regulation of secretion by cell | BP | 9 | 0.00 |
| GO:0009405 | pathogenesis | BP | 36 | 0.00 |
| GO:0071973 | flagellum-dependent cell motility | BP | 23 | 0.00 |
| GO:0009145 | purine nucleoside triphosphate biosynthesis | BP | 9 | 0.00 |
| GO:0009206 | purine ribonucleoside triphosphate biosynthesis | BP | 9 | 0.00 |
| GO:0009205 | purine ribonucleoside triphosphate metabolite | BP | 9 | 0.00 |
| GO:0071453 | cellular response to oxygen levels | BP | 7 | 0.01 |
| GO:0070482 | response to oxygen levels | BP | 7 | 0.01 |
| GO:0045333 | cellular respiration | BP | 18 | 0.01 |
| GO:0098660 | inorganic ion transmembrane transport | BP | 23 | 0.01 |
| GO:0006811 | ion transport | BP | 82 | 0.01 |
| GO:0009201 | ribonucleoside triphosphate biosynthesis | BP | 10 | 0.01 |
| GO:0051051 | negative regulation of transport | BP | 12 | 0.01 |
| GO:0009142 | nucleoside triphosphate biosynthesis | BP | 12 | 0.01 |
| GO:0006928 | movement of cell or subcellular component | BP | 30 | 0.01 |
| GO:0048870 | cell motility | BP | 30 | 0.01 |
| GO:0097588 | type flagellum-dependent cell motility | BP | 23 | 0.01 |
| GO:0001539 | cilium or flagellum-dependent cell motility | BP | 23 | 0.01 |
| GO:0007155 | cell adhesion | BP | 14 | 0.01 |
| GO:0046034 | ATP metabolic process | BP | 16 | 0.01 |
| GO:0009199 | ribonucleoside triphosphate metabolic process | BP | 10 | 0.01 |
| GO:0006112 | energy reserve metabolic process | BP | 4 | 0.01 |
| GO:0019483 | beta-alanine biosynthetic process | BP | 4 | 0.01 |
| GO:0005977 | glycogen metabolic process | BP | 4 | 0.01 |
| GO:0019679 | propionate metabolic process, methylcitrate cycle | BP | 4 | 0.01 |
| GO:0019482 | beta-alanine metabolic process | BP | 4 | 0.01 |
| GO:0098655 | cation transmembrane transport | BP | 28 | 0.01 |
| GO:0006810 | transport | BP | 172 | 0.01 |
| GO:0006071 | glycerol metabolic process | BP | 6 | 0.01 |
| GO:0046903 | secretion | BP | 49 | 0.01 |
| GO:0019646 | aerobic electron transport chain | BP | 5 | 0.01 |
| GO:0006091 | generation of precursor metabolites and energy | BP | 39 | 0.01 |
| GO:0071692 | protein localization to extracellular region | BP | 48 | 0.01 |
| GO:0002790 | peptide secretion | BP | 48 | 0.01 |
| GO:0009306 | protein secretion | BP | 48 | 0.01 |
| GO:0032940 | secretion by cell | BP | 48 | 0.01 |
| GO:0035592 | protein localization to extracellular region | BP | 48 | 0.01 |
| GO:0009144 | purine nucleoside triphosphate biosynthesis | BP | 10 | 0.01 |
| GO:0009083 | branched-chain amino acid catabolic process | BP | 8 | 0.01 |
| GO:0051234 | establishment of localization | BP | 172 | 0.01 |
| GO:0022610 | biological adhesion | BP | 14 | 0.01 |
| GO:0051179 | localization | BP | 172 | 0.01 |
| GO:0034220 | ion transmembrane transport | BP | 33 | 0.02 |
| GO:2001057 | reactive nitrogen species metabolic process | BP | 7 | 0.02 |
| GO:0044550 | secondary metabolite biosynthetic process | BP | 18 | 0.02 |
| GO:0140352 | export from cell | BP | 49 | 0.02 |
| GO:0019400 | alditol metabolic process | BP | 6 | 0.02 |
| GO:0006073 | cellular glucan metabolic process | BP | 6 | 0.02 |
| GO:0046459 | short-chain fatty acid metabolic process | BP | 6 | 0.02 |
| GO:0019541 | propionate metabolic process | BP | 6 | 0.02 |
| GO:0009628 | response to abiotic stimulus | BP | 17 | 0.02 |
| GO:0055072 | iron ion homeostasis | BP | 10 | 0.02 |
| GO:0019748 | secondary metabolic process | BP | 19 | 0.02 |
| GO:0009141 | nucleoside triphosphate metabolic process | BP | 13 | 0.02 |
| GO:0006812 | cation transport | BP | 47 | 0.02 |
| GO:0019563 | glycerol catabolic process | BP | 3 | 0.02 |
| GO:0019405 | alditol catabolic process | BP | 3 | 0.02 |
| GO:0005991 | trehalose metabolic process | BP | 3 | 0.02 |
| GO:0006552 | leucine catabolic process | BP | 4 | 0.02 |
| GO:0019305 | dTDP-rhamnose biosynthetic process | BP | 4 | 0.02 |
| GO:0046383 | dTDP-rhamnose metabolic process | BP | 4 | 0.02 |
| GO:0072337 | modified amino acid transport | BP | 10 | 0.02 |
| GO:0009060 | aerobic respiration | BP | 10 | 0.02 |
| GO:0015672 | monovalent inorganic cation transport | BP | 18 | 0.03 |
| GO:0051049 | regulation of transport | BP | 23 | 0.03 |
| GO:0015990 | electron transport coupled proton transport | BP | 6 | 0.03 |
| GO:0015988 | energy coupled proton transmembrane transport | BP | 6 | 0.03 |
| GO:0044042 | glucan metabolic process | BP | 6 | 0.03 |
| GO:0009061 | anaerobic respiration | BP | 6 | 0.03 |
| GO:0055085 | transmembrane transport | BP | 102 | 0.03 |
| GO:0071978 | flagellum-dependent swarming motility | BP | 14 | 0.03 |
| GO:0055114 | oxidation-reduction process | BP | 49 | 0.03 |
| GO:0051606 | detection of stimulus | BP | 9 | 0.03 |
| GO:0055076 | transition metal ion homeostasis | BP | 11 | 0.03 |
| GO:0007165 | signal transduction | BP | 46 | 0.03 |
| GO:0097435 | supramolecular fiber organization | BP | 5 | 0.03 |
| GO:0055065 | metal ion homeostasis | BP | 11 | 0.04 |
| GO:0032879 | regulation of localization | BP | 32 | 0.04 |
| GO:0042908 | xenobiotic transport | BP | 8 | 0.04 |
| GO:0015711 | organic anion transport | BP | 36 | 0.04 |
| GO:0071806 | protein transmembrane transport | BP | 41 | 0.05 |
| GO:0071702 | organic substance transport | BP | 111 | 0.05 |
| GO:0016054 | organic acid catabolic process | BP | 54 | 0.05 |
| GO:0005576 | extracellular region | CC | 23 | 0.00 |
| GO:0009424 | bacterial-type flagellum hook | CC | 8 | 0.00 |
| GO:0019867 | outer membrane | CC | 44 | 0.00 |
| GO:0009279 | cell outer membrane | CC | 37 | 0.01 |
| GO:0045263 | proton-transporting ATP synthase complex | CC | 4 | 0.01 |
| GO:0033177 | proton-transporting two-sector ATPase complex | CC | 4 | 0.01 |
| GO:0016020 | membrane | CC | 230 | 0.01 |
| GO:0042995 | cell projection | CC | 11 | 0.02 |
| GO:0045261 | proton-transporting ATP synthase complex | CC | 5 | 0.02 |
| GO:0046930 | pore complex | CC | 5 | 0.02 |
| GO:0033178 | proton-transporting two-sector ATPase complex | CC | 5 | 0.02 |
| GO:0070469 | respirasome | CC | 5 | 0.02 |
| GO:0030694 | bacterial-type flagellum basal body, rod | CC | 3 | 0.02 |
| GO:0009325 | nitrate reductase complex | CC | 3 | 0.02 |
| GO:0009289 | pilus | CC | 10 | 0.02 |
| GO:0070069 | cytochrome complex | CC | 6 | 0.03 |
| GO:0045239 | tricarboxylic acid cycle enzyme complex | CC | 5 | 0.03 |
| GO:0005615 | extracellular space | CC | 9 | 0.04 |
| GO:0044096 | type IV pilus | CC | 6 | 0.04 |
| GO:0038023 | signaling receptor activity | MF | 22 | 0.00 |
| GO:0004888 | transmembrane signaling receptor activity | MF | 10 | 0.00 |
| GO:0015075 | ion transmembrane transporter activity | MF | 78 | 0.00 |
| GO:0046933 | proton-transporting ATP synthase activity | MF | 7 | 0.00 |
| GO:0005261 | cation channel activity | MF | 8 | 0.00 |
| GO:0008940 | nitrate reductase activity | MF | 5 | 0.00 |
| GO:0016675 | oxidoreductase activity | MF | 12 | 0.00 |
| GO:0004130 | cytochrome-c peroxidase activity | MF | 6 | 0.01 |
| GO:0015252 | proton channel activity | MF | 7 | 0.01 |
| GO:0008324 | cation transmembrane transporter activity | MF | 50 | 0.01 |
| GO:0060089 | molecular transducer activity | MF | 34 | 0.01 |
| GO:0004129 | cytochrome-c oxidase activity | MF | 11 | 0.01 |
| GO:0015002 | heme-copper terminal oxidase activity | MF | 11 | 0.01 |
| GO:0016676 | oxidoreductase activity | MF | 11 | 0.01 |
| GO:0004553 | hydrolase activity | MF | 10 | 0.01 |
| GO:0015453 | oxidoreduction | MF | 4 | 0.01 |
| GO:0022803 | passive transmembrane transporter activity | MF | 24 | 0.01 |
| GO:0015267 | channel activity | MF | 24 | 0.01 |
| GO:0015318 | inorganic molecular entity transmembrane transport | MF | 65 | 0.01 |
| GO:0005216 | ion channel activity | MF | 9 | 0.01 |
| GO:0016682 | oxidoreductase activity | MF | 5 | 0.01 |
| GO:0046906 | tetrapyrrole binding | MF | 31 | 0.01 |
| GO:0015077 | inorganic cation transmembrane transport | MF | 29 | 0.01 |
| GO:0015078 | proton transmembrane transporter activity | MF | 23 | 0.01 |
| GO:0072349 | modified amino acid transmembrane transport | MF | 9 | 0.02 |
| GO:0020037 | heme binding | MF | 29 | 0.02 |
| GO:0004601 | peroxidase activity | MF | 14 | 0.02 |
| GO:0016684 | oxidoreductase activity | MF | 15 | 0.02 |
| GO:0022890 | inorganic cation transmembrane transport | MF | 36 | 0.02 |
| GO:0003849 | 3-deoxy-7-phosphoheptulonate synthase activity | MF | 5 | 0.02 |
| GO:0016652 | oxidoreductase activity | MF | 5 | 0.02 |
| GO:0015343 | siderophore transmembrane transport | MF | 13 | 0.02 |
| GO:0022857 | transmembrane transporter activity | MF | 171 | 0.02 |
| GO:0009486 | cytochrome bo3 ubiquinol oxidase activity | MF | 3 | 0.02 |
| GO:0008750 | NAD(P)+ transhydrogenase (AB-specific) activity | MF | 3 | 0.02 |
| GO:0016803 | ether hydrolase activity | MF | 3 | 0.02 |
| GO:0008556 | potassium transmembrane transport | MF | 3 | 0.02 |
| GO:0003826 | alpha-ketoacid dehydrogenase activity | MF | 3 | 0.02 |
| GO:0050622 | glycine dehydrogenase (cyanide-forming) activity | MF | 3 | 0.02 |
| GO:0019154 | glycolate dehydrogenase activity | MF | 3 | 0.02 |
| GO:0008827 | cytochrome o ubiquinol oxidase activity | MF | 3 | 0.02 |
| GO:0003985 | acetyl-CoA C-acetyltransferase activity | MF | 4 | 0.02 |
| GO:0016453 | C-acetyltransferase activity | MF | 4 | 0.02 |
| GO:0033818 | beta-ketoacyl-acyl-carrier-protein synthase III | MF | 4 | 0.02 |
| GO:0005215 | transporter activity | MF | 173 | 0.02 |
| GO:0030151 | molybdenum ion binding | MF | 6 | 0.03 |
| GO:0071949 | FAD binding | MF | 12 | 0.03 |
| GO:0016705 | oxidoreductase activity | MF | 21 | 0.03 |
| GO:0009055 | electron transfer activity | MF | 39 | 0.03 |
| GO:0016758 | transferase activity | MF | 13 | 0.03 |
| GO:0019829 | ATPase-coupled cation transmembrane transport | MF | 6 | 0.04 |
| GO:0016661 | oxidoreductase activity | MF | 8 | 0.04 |
| GO:0042910 | xenobiotic transmembrane transport | MF | 16 | 0.04 |
| GO:0010340 | carboxyl-O-methyltransferase activity | MF | 4 | 0.04 |
| GO:0004049 | anthranilate synthase activity | MF | 4 | 0.04 |
| GO:0015344 | siderophore uptake transmembrane transport | MF | 12 | 0.05 |
| GO:1901682 | sulfur compound transmembrane transport | MF | 12 | 0.05 |
